# Testing the Feasibility and Acceptability of Using an Artificial Intelligence Chatbot to Promote HIV Testing and Pre-Exposure Prophylaxis in Malaysia: Mixed Methods Study

**DOI:** 10.2196/52055

**Published:** 2024-01-26

**Authors:** Min Hui Cheah, Yan Nee Gan, Frederick L Altice, Jeffrey A Wickersham, Roman Shrestha, Nur Afiqah Mohd Salleh, Kee Seong Ng, Iskandar Azwa, Vimala Balakrishnan, Adeeba Kamarulzaman, Zhao Ni

**Affiliations:** 1 Department of Medicine Faculty of Medicine University of Malaya Kuala Lumpur Malaysia; 2 Department of Social and Preventive Medicine Faculty of Medicine University of Malaya Kuala Lumpur Malaysia; 3 Section of Infectious Disease Department of Internal Medicine Yale School of Medicine New Haven, CT United States; 4 Division of Epidemiology of Microbial Diseases Yale School of Public Health New Haven, CT United States; 5 Center for Interdisciplinary Research on AIDS (CIRA) Yale University New Haven, CT United States; 6 Centre of Excellence for Research in AIDS (CERiA) Faculty of Medicine University of Malaya Kuala Lumpur Malaysia; 7 Department of Allied Health Sciences University of Connecticut Storrs, CT United States; 8 Department of Information Systems Faculty of Computer Science and Information Technology University of Malaya Kuala Lumpur Malaysia; 9 Monash University Malaysia Kuala Lumpur Malaysia; 10 School of Nursing Yale University Orange, CT United States

**Keywords:** artificial intelligence, acceptability, chatbot, feasibility, HIV prevention, HIV testing, men who have sex with men, MSM, mobile health, mHealth, preexposure prophylaxis, PrEP, mobile phone

## Abstract

**Background:**

The HIV epidemic continues to grow fastest among men who have sex with men (MSM) in Malaysia in the presence of stigma and discrimination. Engaging MSM on the internet using chatbots supported through artificial intelligence (AI) can potentially help HIV prevention efforts. We previously identified the benefits, limitations, and preferred features of HIV prevention AI chatbots and developed an AI chatbot prototype that is now tested for feasibility and acceptability.

**Objective:**

This study aims to test the feasibility and acceptability of an AI chatbot in promoting the uptake of HIV testing and pre-exposure prophylaxis (PrEP) in MSM.

**Methods:**

We conducted beta testing with 14 MSM from February to April 2022 using Zoom (Zoom Video Communications, Inc). Beta testing involved 3 steps: a 45-minute human-chatbot interaction using the think-aloud method, a 35-minute semistructured interview, and a 10-minute web-based survey. The first 2 steps were recorded, transcribed verbatim, and analyzed using the Unified Theory of Acceptance and Use of Technology. Emerging themes from the qualitative data were mapped on the 4 domains of the Unified Theory of Acceptance and Use of Technology: performance expectancy, effort expectancy, facilitating conditions, and social influence.

**Results:**

Most participants (13/14, 93%) perceived the chatbot to be useful because it provided comprehensive information on HIV testing and PrEP (*performance expectancy*). All participants indicated that the chatbot was easy to use because of its simple, straightforward design and quick, friendly responses (*effort expectancy*). Moreover, 93% (13/14) of the participants rated the overall chatbot quality as high, and all participants perceived the chatbot as a helpful tool and would refer it to others. Approximately 79% (11/14) of the participants agreed they would continue using the chatbot. They suggested adding a local language (ie, Bahasa Malaysia) to customize the chatbot to the Malaysian context (*facilitating condition*) and suggested that the chatbot should also incorporate more information on mental health, HIV risk assessment, and consequences of HIV. In terms of *social influence*, all participants perceived the chatbot as helpful in avoiding stigma-inducing interactions and thus could increase the frequency of HIV testing and PrEP uptake among MSM.

**Conclusions:**

The current AI chatbot is feasible and acceptable to promote the uptake of HIV testing and PrEP. To ensure the successful implementation and dissemination of AI chatbots in Malaysia, they should be customized to communicate in Bahasa Malaysia and upgraded to provide other HIV-related information to improve usability, such as mental health support, risk assessment for sexually transmitted infections, AIDS treatment, and the consequences of contracting HIV.

## Introduction

### Background

HIV continues to be a global health concern causing approximately 630,000 deaths yearly worldwide [1]. In 2019, approximately 62% of the new HIV infections among adults worldwide occurred within key populations and their sexual partners [2]. Men who have sex with men (MSM) accounted for 23% of new infections of HIV, which was much higher than the percentage of new infections in other key populations, such as people who used drugs (10%), sex workers (8%), and transgender people (2%) in 2019 [3]. Malaysia is a Southeast Asian country with a population of 33.5 million, with 1 in 5 MSM living with HIV [4]. Over the past 2 decades, the mode of HIV transmission in Malaysia has shifted from needle sharing to sexual transmission, particularly among MSM [5].

HIV testing is a prerequisite to effective HIV prevention and early treatment initiation [6]; people at risk for HIV or seropositive individuals need to be tested for HIV before being linked to health care services [7]. Despite the importance of HIV testing, it is disproportionately lower among MSM in Malaysia [8]. New HIV testing guidelines recommend that MSM at high risk for HIV should be tested every 3 to 6 months, but most MSM in Malaysia do not test optimally. Studies in Malaysia have found that only 9.5% of MSM tested more than once a year. In Malaysia, engaging in same-sex sexual behavior is prohibited by both secular and Sharia laws, leading to significant levels of stigma and discrimination within society [9]. As a result, many MSM may be hesitant or unwilling to engage with health care providers and outreach workers. Therefore, designing new strategies to promote HIV testing among MSM in Malaysia is urgently needed [10].

Using portable electronic devices with software programs to deliver health care services and manage patient information is known as mobile health (mHealth) [11]. mHealth interventions could reduce barriers to HIV testing for MSM by reducing in-person contact and offering internet-based platforms for HIV testing [12,13]. Studies have demonstrated that mHealth interventions using smartphones and apps could increase the uptake of HIV testing while protecting the privacy of MSM [14-16], and MSM in Malaysia have a high acceptance of the use of mHealth for HIV testing and prevention [13,17,18]. Recent breakthroughs in artificial intelligence (AI) and machine learning can potentially automate and scale up these mHealth interventions through chatbots, a computer program that can mimic human conversation [19]. However, leveraging chatbot technology to promote HIV testing and prevention is in its infancy [15,20]. Although chatbot technology holds immense potential to prevent HIV, a lack of research in this field undermines its significance. The creation of ChatGPT has brought attention to the significance of studying chatbot technology for health care.

Our team has conducted formative research to understand HIV prevention chatbots in Malaysia and has identified the perceived benefits, limitations, and preferred features of AI chatbots for HIV testing and prevention among MSM [13]. On the basis of the study findings, we developed an HIV prevention AI chatbot prototype named Haris (a common Malaysian name) and a website called MYHIV365 (*MY* symbolizes Malaysia, *HIV* implies health care services aimed at preventing HIV, and *365* indicates the services are available every day of the year). Haris was hosted on MYHIV365 and could provide information on the 3 themes most needed by MSM: HIV testing, mental health, and pre-exposure prophylaxis (PrEP). PrEP is a highly effective HIV prevention method that involves the use of antiretroviral medication by at-risk individuals to prevent getting HIV from sex or injection drug use. Haris imitates human intelligence and can interact with users to provide support, including ordering free HIV self-testing kits, screening for depression, and recommending MSM-friendly clinics where individuals can get tested for HIV and receive PrEP.

### Objectives

Despite the meticulous design and alpha testing (internal testing) of Haris among professors, experts, and community advisory board members, its feasibility and acceptability in preventing HIV among MSM is still unknown. Therefore, we conducted beta testing (testing in a real-world environment by actual users) of Haris among 14 MSM in Malaysia to address this knowledge gap. Specifically, we examined the use of the AI chatbot for delivering health information and improving linkage to HIV testing, PrEP, and care. We also investigated key strategies to refine the feasibility and acceptability of the AI chatbot in this study.

## Methods

### Study Design and Participants

Beta testing of the AI chatbot prototype was conducted with 14 MSM by an experienced qualitative interviewer (ZN) with expertise in chatbot development and HIV prevention in Malaysia and 4 trainees in the Malaysian Implementation Science Training program (Fogarty International Center, D43TW011324). Participants were recruited in Malaysia from February to April 2022 via social networking apps commonly used by MSM, including Grindr, Hornet, Blued, and WhatsApp. The procedures for participant recruitment have been published elsewhere [13]. A web-based screener including questions on demographic characteristics and HIV prevention practices was used. The eligibility criteria included (1) self-identification as a cisgender man, (2) age ≥18 years, (3) condomless sex with another man in the past 6 months, and (4) being HIV negative or of unknown status.

**Figure 1:**
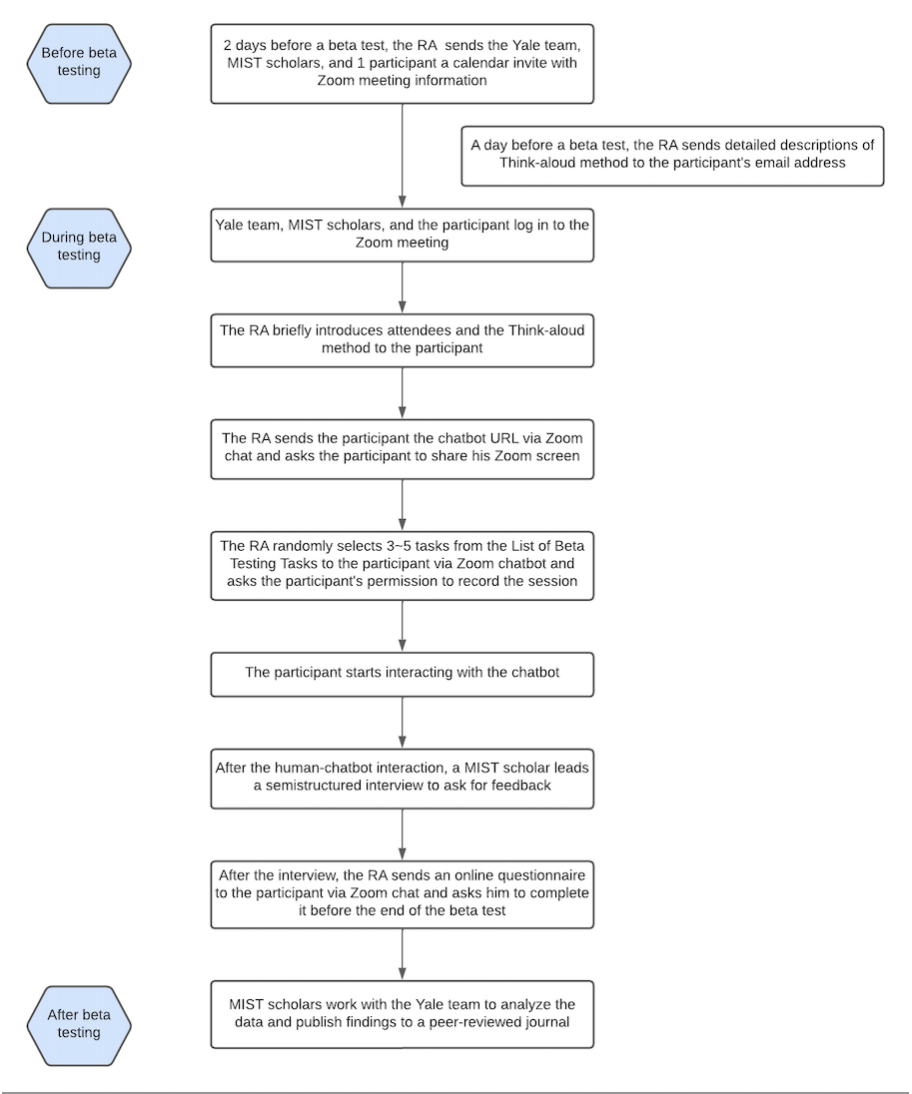
Study procedure. MIST: Malaysian Implementation Science Training; RA: research assistant.

Each beta test involved the following three steps: (1) a 45-minute human-chatbot interaction using the think-aloud method [21]; (2) a 35-minute semistructured interview; and (3) a 10-minute web-based survey. The first 2 steps were conducted via Zoom (Zoom Video Communications, Inc), recorded, and transcribed verbatim. Specifically, 2 days before the test, a research assistant sent a calendar invite with Zoom meeting information to the interviewer and participant. One day before beta testing, the research assistant emailed the participant a detailed description of the human-chatbot interaction (Multimedia Appendix 1). During the human-chatbot interaction, participants were asked to share their screen via Zoom and access the chatbot through a URL sent by the research assistant. After the participants obtained access to the chatbot, the research assistant randomly selected 3 to 5 tasks from the list of beta testing tasks (Multimedia Appendix 2) and asked the participants to complete them through the chatbot. Some examples of the tasks include “find a clinic that can provide HIV testing service in Kuala Lumpur” and “find out the common symptoms of depression through the chatbot.” The study procedure is described in Figure 1.

After the human-chatbot interaction, we conducted a semistructured interview (Multimedia Appendix 3 [22]) soliciting participants’ feedback on two themes: (1) experience navigating the chatbot and (2) how the chatbot should be made available to a wider audience. During the interview, participants were asked several questions regarding their experience with the chatbot, such as “How was your experience with the AI chatbot?”, “What feature of the AI chatbot do you like the most?”, and “What information needs to be added to the AI chatbot to increase its popularity among MSM?” After the interviews concluded, the participants were provided with a survey link to assess the feasibility and acceptability of the AI chatbot. The feasibility of the chatbot was measured through 4 outcomes, including participants’ ratings of the chatbot’s quality, satisfaction, intention to continue using the chatbot, and willingness to refer it to others. The outcomes were measured using a 10-point rating scale, with higher scores indicating more favorable outcomes (Multimedia Appendix 4). For example, participants’ satisfaction with the chatbot was measured by using the question, “How satisfied were you with the experience of interacting with the chatbot?” The score of “0” stands for not satisfactory at all and “10” stands for extremely satisfactory. The acceptability of the chatbot was measured using the standardized System Usability Scale [23] and an adjusted Chatbot Usability Scale [24]. The combination of the 2 scales provided a comprehensive evaluation of the acceptability of our chatbot.

### Ethical Considerations

The participants provided electronic consent before initiating the beta testing. This study was approved by the institutional review board of Yale University (approval #2000027864) and Medical Research Ethics Committee of the University of Malaya (approval #2021112-10729). This research was conducted in accordance with the ethical standards of the 1964 Helsinki Declaration and its later amendments or comparable ethical standards.

### Conceptual Framework for Analysis

The Unified Theory of Acceptance and Use of Technology (UTAUT) was used as a conceptual framework to guide the analysis of the experience of MSM using the AI chatbot for HIV testing and prevention in Malaysia. UTAUT consists of four domains: (1) performance expectancy, (2) effort expectancy, (3) facilitating conditions, and (4) social influence [25]. The definitions of these 4 domains have been published elsewhere [13]. UTAUT was chosen for the following reasons. First, this AI chatbot was developed based on the findings from a formative research project that was analyzed using UTAUT [13]. Therefore, using the same theory, we can compare the results of the 2 studies on the 4 domains and are more likely to find out the feasibility and acceptability of the AI chatbot. Second, UTAUT emphasizes user-centered perspective, which allows researchers to assess the acceptance of the AI chatbot from the users’ perception. Third, UTAUT has been extensively used to identify users’ acceptance of technology and was reported to be effective and of high validity [26,27].

### Analyses

All transcripts were cross-checked for accuracy and completeness by 7 researchers (MHC, YNG, NAMS, KSN, ZN, and 2 research assistants). Each of the 7 researchers independently coded 2 transcripts using NVivo 10 software (QSR International), compiled codes, and mapped the emerging themes from the qualitative data on the 4 domains of UTAUT, including performance expectancy, effort expectancy, facilitating conditions, and social influence. Discrepancies in codes and themes were addressed in group discussions where there was discordance in coding. We ceased the qualitative analysis when the results reached saturation, and no new themes emerged. The participants’ quotes are presented throughout the results with additional quotes given in Multimedia Appendix 5. Quantitative data from the survey were analyzed using SAS (version 9.4; SAS Institute) and are presented as descriptive statistics.

## Results

### Participant Characteristics

The 14 participants were on average in their mid-20s (mean 25.6, SD 4.2 years), and most of them (13/14, 93%) used smartphones as the primary means to access the internet. Most participants (10/14, 71%) were Malay, followed by Chinese (3/14, 21%) and Indian (1/14, 7%). About one-third of the participants (5/14, 36%) had taken PrEP previously, and only 14% (2/14) of them were currently taking PrEP. The demographic characteristics are summarized in Table 1.

**Table 1:** Participant demographic details (N=14).

Characteristics			Values
Age (y), mean (SD)			25.6 (4.2)
**Ethnicity, n (%)**			
	Malay	10 (71)	
	Chinese	3 (21)	
	Indian	1 (7)	
**Sexual orientation, n (%)**			
	Bisexual	3 (21)	
	Gay	11 (79)	
**Employment status, n (%)**			
	Student	6 (43)	
	Working full time	8 (57)	
**Highest level of education, n (%)**			
	Diploma or bachelor degree	8 (57)	
	Master degree or PhD	3 (21)	
	Secondary school	3 (21)	
**Average monthly income (MYR^a^; 1 MYR=US $0.21), n (%)**			
	<2000	6 (43)	
	2000-4000	5 (36)	
	>4000	3 (21)	
**Daily access to the internet, n (%)**			
	Yes	14 (100)	
**Primary device for accessing the internet, n (%)**			
	Smartphone	13 (93)	
	Laptop computer	1 (7)	
**Had ever taken PrEP^b^, n (%)**			
	Yes	5 (36)	
	No	9 (64)	
**Currently taking PrEP, n (%)**			
	Yes	2 (14)	
	No	12 (86)	

^a^MYR: Malaysian Ringgit.

^b^PrEP: pre-exposure prophylaxis.

### Feasibility

**Figure 2:**
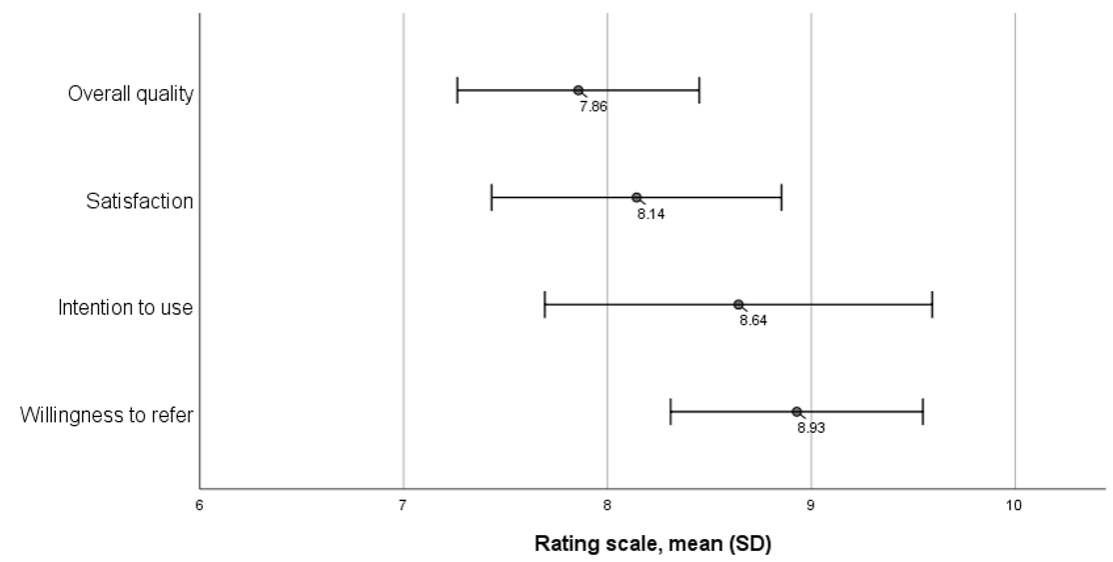
Feasibility ratings of the chatbot.

The mean scores on the 4 metrics of the feasibility of the chatbot, overall quality, satisfaction, intention to continue using, and willingness to refer to others were 7.86 (SD 1.03), 8.14 (SD 1.23), 8.64 (SD 1.65), and 8.93 (SD 1.07), respectively, on a scale from 0 to 10 (Figure 2).

### Acceptability

**Figure 3:**
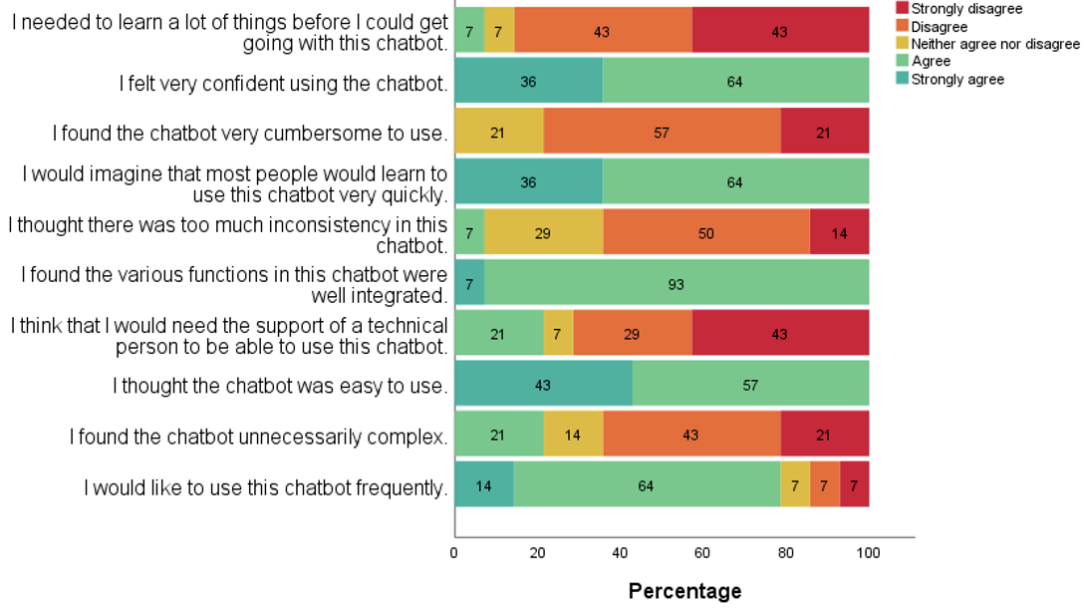
System Usability Scale outcomes.

The participants found the chatbot acceptable, as it was perceived as easy to navigate and capable of providing valuable information (Multimedia Appendix 6). Specifically, all participants (14/14, 100%) expressed confidence in using the chatbot, believing that others could also quickly master its use (Figure 3). The overall mean (SD) score of the System Usability Scale was 76.07 (SD 8.19), which is greater than the recommended acceptable cutoff score of 68 [23].

**Figure 4:**
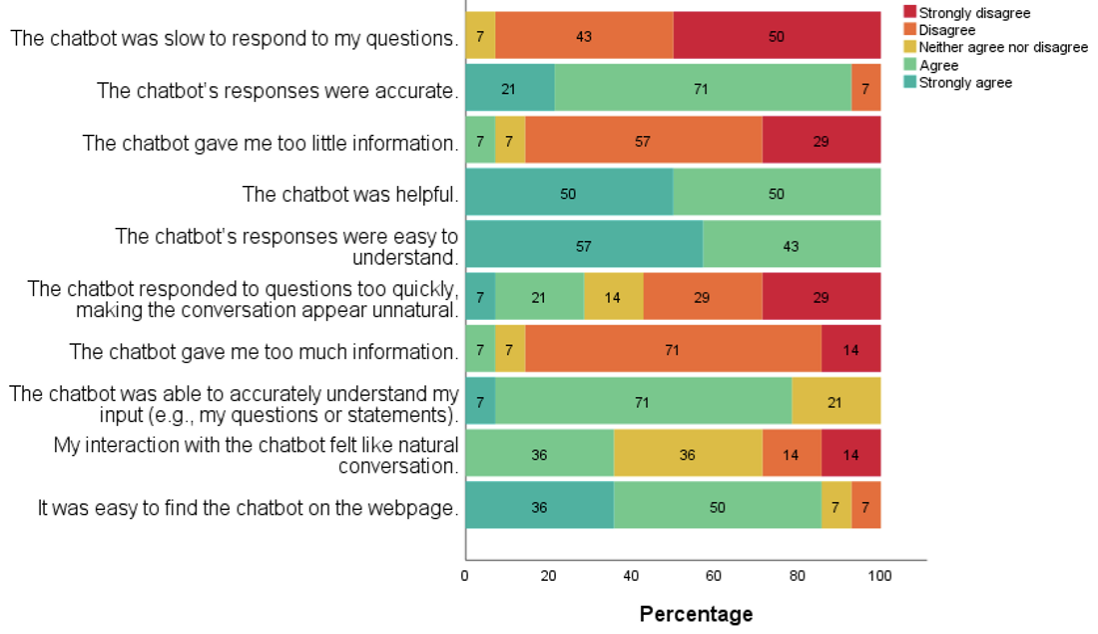
Adjusted Chatbot Usability Scale outcomes.

Moreover, 78% (11/14) of the participants agreed that the chatbot could understand their inputs accurately (Figure 4). However, only 36% (5/14) of the participants agreed that their interaction with the chatbot felt like a natural conversation.

In addition to measuring feasibility and acceptability, we summarized the study findings based on the 4 domains of UTAUT: performance expectancy, effort expectancy, facilitating conditions, and social influence.

### Performance Expectancy

#### Overall Perception

Participants responded positively about the performance of the AI chatbot. Quantitative data analysis revealed that all participants (14/14, 100%) perceived the chatbot as a helpful tool, they would refer it to others, and 93% (13/14) of them highly rated the overall quality of the chatbot. These results were consistent with the qualitative finding that participants were satisfied with the information provided by the chatbot and deemed it a trustable source.

#### Contributors to Positive Performance Expectancy

The chatbot served as a reliable source of information. Participants expressed their trust in this chatbot as they believed it was developed to help people learn more about HIV. For instance, one participant stated that he trusted the chatbot more than other internet-based platforms as the following:

I can actually trust, trust this chatbot more than I can trust the Internet.

Interview E

Most participants expressed that the information provided by the chatbot was comprehensive and satisfactory. The same participant highlighted the following:

...everything is there, everything is informative...

Interview E

Participants described the chatbot’s function of ordering free HIV self-test kits as one of the most useful functions. The simple and straightforward instructions from the chatbot significantly encouraged participants to perform HIV self-testing and helped prevent misuse of the self-test kit. A participant elaborated on the following:

...it never crossed my mind that you can do HIV self-test just using it (test stick) over the gums and without blood...

Interview G

In addition to the positive feedback on the chatbot’s ability to order free HIV self-test kits, participants also expressed appreciation for the information provided by the chatbot on MSM-friendly local clinics where they could test for HIV and receive PrEP consultation. For example, the participant stated the following:

That was beyond amazing...they give [me] the addresses, contact numbers. So, I would say, if a person really needs to do the [HIV] testing, essential information like that would be very useful, so I think it’s more than helpful. The options [of HIV testing clinics] are not just [limited to] one or two [clinics], you know, so that’s good.

Interview G

The other participants stressed that the chatbot’s features related to HIV self-testing and venue-based HIV testing were complementary and appreciated having access to both options through the chatbot. Although performing self-testing at home may be convenient, it may lack the human interaction that some MSM need for support during the testing process. By offering both self-testing and venue-based testing options, the chatbot gave MSM the flexibility to choose the option that best suited their individual needs and preferences. For example, a participant emphasized that the chatbot could enable his MSM friends to conduct HIV self-testing and then see a health care professional for advice on complex and sensitivity issues:

...the reason why [they] went to a health care facility was [that they] can have someone tell them that you know, “Being tested positive for HIV [was] not the end of the world. To reduce the [HIV] symptoms, to reduce the [HIV] effect, to reduce [their HIV] viral load... [they] can still live a normal life and so on,” which might be something that the self-test kits [were] lacking.

Interview C

Along with the positive feedback on the chatbot’s features relevant to HIV testing, the participants also expressed their favorable feelings toward the PrEP information provided by the chatbot. For example, a participant mentioned that the chatbot could send him introductory information to allow him to comfortably assess his risk level and help him decide if he needed to take PrEP. Another participant reiterated the first participant’s point by emphasizing the difficulties that MSM in determining if they should take PrEP, stating the following:

A lot of people [MSM] are always asking themselves, “Should I get PrEP? Am I at risk? Do I need to take PrEP as a precautionary measure?”... So these are [questions] that MSM usually a bit too scared to ask the doctors.

Interview C

#### Major Concerns

Participants suggested that the chatbot would be improved if it could provide more information and resources relevant to mental health, as mental health issues were the prominent problems that MSM faced in Malaysia. Participants wanted the chatbot to provide information on strategies for managing stress, statistics about depression among MSM, peer consultation for depression, and professional health care services to prevent and treat depression. The participants also highlighted that the MYHIV365 website, where the chatbot was embedded, should provide more resources related to mental health. For example, one participant described this problem as follows:

The website did not have links to any information regarding mental health issues, and that is a glaring issue for it to be left out like that.

Interview A

The same statement was echoed by another participant, who stressed the following:

I just find that for the mental health, it’s kind of short.

Interview G

#### Relevant Features Suggested by Participants That Are Needed to Improve the Chatbot

Although the chatbot made it easy for participants to receive an HIV self-test kit, one participant suggested that a step-by-step video demonstrating how to use the kit could be helpful for MSM who were testing for HIV. The participant stated the following:

If the self-test kit has got like an instructional video or something like that to kind of guide the users along the way of getting [themselves] tested, I think that’d be great because not everyone knows how to use a kit successfully.

Interview C

Although the AI chatbot was developed primarily for HIV prevention and to assist with HIV testing and access to PrEP, the participants pointed out that some participants may test positive for HIV and would benefit from learning more about accessing HIV care and related antiretroviral therapy services. In addition, participants suggested that the chatbot should provide more information about antiretroviral therapy so that users could better manage HIV by knowing potential drug interactions and side effects. The participants also recommended providing more information about high-risk behaviors and sexually transmitted diseases to help increase awareness about HIV and sexually transmitted diseases among MSM. One participant stated the following:

I think [providing more information about] HIV treatment would [be] very helpful because those who might be exposed to HIV would definitely want to know what the treatment is all about.

Interview E

This participant’s statement was echoed by another participant, who stated the following:

...HIV and STDs...[are] not the same, but...I thought [they were] the same...I thought HIV and STDs were not curable...so I think it will be great if you can add STDs [to the chatbot].

Interview E

### Effort Expectancy

#### Overall Perception

All surveyed participants (14/14, 100%) agreed that the chatbot was easy to use, and 86% (12/14) of the participants were satisfied with the chatbot. In the qualitative interviews, participants reported consistent feedback that the chatbot was user friendly and convenient to use, and they were satisfied with the chatbot because of its simple, straightforward design and quick, friendly responses. However, they were concerned about the technical issues, including the address input and text alignments (refer to the *Major Concerns* section). The participants also felt that tailoring the chatbot to the local context and adding a “human touch” would be helpful.

#### Positive Contributors to Low Effort Expectancy

Many participants expressed satisfaction with the chatbot because of its prompt response, expert information, and plain interface. Two participants commented the following:

...white and blue colors [are] neutral, and it [the chatbot] takes into account [of] color blindness as well, so that’s great.

Interview C

...[I] got a quick response [from the chatbot].

Interview I

The individualized and user-centered features of the chatbot, which cater to users with different levels of communication skills, were highlighted among the participants. For instance, one participant stated that the chatbot offered an ideal platform for MSM who are less comfortable interacting with others. A participant stated the following:

As we all know, some of us didn’t have the skills to communicate, so I think...[the] chatbot… will definitely help. I think it was great.

Interview E

Moreover, participants thought the chatbot was useful as it facilitated them to obtain culturally tailored health information. The chatbot met users’ needs by providing a menu of options for users to choose from. Compared with obtaining health information in clinical settings in Malaysia, the chatbot was much simpler. A participant elaborated on the following:

When [the chatbot] come[s] up with three options like that, I can explore myself...I would say that [the chatbot] is more precise; it gets to the point directly.

Interview G

The health intervention being tailored to the local setting was highly valued by the participants. Responses from the AI chatbot that contained localized features, specifically the use of “Manglish,” a less formal form of Malaysian English, were appreciated by several participants. The feature of “Manglish,” which was not in the standardized form of English, has added a local flavor to the AI chatbot, which some participants found amusing. A participant stated the following:

The impression that this chatbot...probably comes from America. It’s in English, so the moment it puts up a Malaysian style saying “Boleh”... I’m very amused with this [style].

Interview G

#### Major Concerns

Some participants spoke about the difficulty in filling in their home addresses using the current prompts on the AI chatbot when they needed to order an HIV self-test kit; the chatbot required a step-by-step input of addresses, which was counterintuitive and inefficient. Participants preferred the standard address format in Malaysia over the step-by-step input format, in which incomplete addresses would triage further prompts to ask participants to refill the HIV self-test order. For example, one participant stated the following:

In Malaysia, we don’t use the term “line address” or “street address”. We usually enter the full address with the postcode and then the city and state. The one on the chatbot seems to be how addresses are filled in the United States. That part needs to be tweaked slightly based on Malaysian cities.

Interview C

In addition, participants expressed that the address of the clinics provided by the chatbot needed to be tailored to Malaysian culture. For example, the district options may only be needed for certain states in Malaysia. A participant stated the following:

I think depends on the size of the state...we don’t have to call out (provide choices for) all the districts because Perlis is already small enough, and I think...people can just go easily from one place to another in Perlis. But if...it is a big state...we need to divide it using district.

Interview I

#### Relevant Features Suggested by Participants That Are Needed to Improve the Chatbot

Although participants were satisfied with the AI chatbot, 2 participants suggested that the chatbot’s interface could be improved by adding more spaces between sentences, and the alignment of sentences should be adjusted to make the chatbot look more professional. Two participants described the following:

...everything is tightly together with very little gap...there should be proper spacing...

Interview L

The text is not properly centered in some of the boxes, and I feel like it could [be] a better design to make it look more professional.

Interview A

The use of English as the only language of the AI chatbot was perceived by participants as a barrier to implementing the chatbot in Malaysia. Although all participants were proficient in English, concerns arose for the communities where English was not widely spoken. Participants suggested that the chatbot should be able to communicate in Bahasa Malaysia or Mandarin, given that the 2 languages are widely spoken in Malaysia. A participant stated the following:

...perhaps to have another option of language...I think that would be able to cover more people within the local population.

Interview C

Adding a “human touch” to the chatbot can create a more engaging and user-friendly experience for the users interacting with the chatbot. The participant described the following:

...ideally, we would want [the chatbot’s response] to be as human as possible, and not so robotic in its responses...a nice touch to make someone feel slightly comfortable.

Interview C

### Facilitating Conditions

#### Overall Perception

Participants reported 2 major facilitating conditions for the use of the AI chatbot. First, the social distancing policy adopted by the Malaysian government during the COVID-19 pandemic significantly increased the use of internet-based platforms to seek health information and consult about health issues among MSM. The participants expressed that the AI chatbot was a novel tool to promote HIV testing and prevention among MSM in Malaysia. A participant highlighted the convenience of using the chatbot as an alternative to meeting health care workers during the COVID-19 pandemic as follows:

...because now it’s COVID, everyone is doing it in IT (information technology) format. Having an AI chatbot is definitely much more convenient than meeting people...

Interview G

Second, the AI chatbot’s capability of referring webpages to participants where they could find mental health information, community support, and counselors was a significant facilitator for them to accept the chatbot. Many participants stated that it was much easier to obtain information through the links provided by the chatbot than searching for information via websites or mobile apps. A participant stated the following:

When you interact with it (the chatbot), it throws out links to you. It’s easier to navigate to the particular links from there.

Interview G

#### Relevant Features Suggested by Participants That Are Needed to Improve the Chatbot

Participants suggested that the chatbot could be promoted through social media platforms, such as Facebook, Twitter, Instagram, YouTube, TikTok, and Telegram because these platforms were widely used by MSM as sources of information. Among all social networking apps, participants stated that Twitter was the best platform to advertise the AI chatbot because Twitter enabled users to post clickable links in the comments section where other users could access the chatbot. Participants further reported that Telegram was a more suitable platform for hosting the chatbot than the most popular text messaging app in Malaysia, WhatsApp. Telegram offers a more private and secure environment for MSM to ask questions or express concerns about HIV and AIDS. Participants also suggested that building a trustable relationship between the AI chatbot and the MSM community is key to implementing the AI chatbot in Malaysia. Given that there were many scams through pop-up advertisements on social media platforms, a participant described the following imagined scenario:

...if we play our Facebook, Twitter, Instagram, or YouTube, there are always mini advertisements, so who knows, [whether we] can add this [AI chatbot]?...I need to know about this [chatbot], and I hope this [chatbot] is not a scam.

Interview B

### Social Influence

In terms of *social influence*, the chatbot was perceived as helpful in avoiding HIV stigma and thus could increase the HIV testing rate and PrEP uptake frequency. Quantitative data analysis found that 79% (11/14) of the participants agreed to continue using the chatbot. During the interviews, these participants reported that societal stigma and discrimination related to HIV and AIDS would make them more likely to use the chatbot. They expressed discomfort in asking people questions about HIV and AIDS as they were afraid of encountering stigma and negative attitudes from others. MSM often preferred to seek information through internet-based platforms, and the chatbot was helpful, particularly for people living in small social circles. A participant elaborated on the following:

...this topic [HIV] is quite sensitive to most people, it will create like a negative energy around you...personally I don’t go and ask people what HIV is, I will search myself maybe on the Internet...

Interview E

The societal stigma and discrimination toward HIV and AIDS also facilitated participants to select HIV self-testing at home rather than testing in a clinical setting. Many participants appreciated that the chatbot offered them an opportunity to receive free HIV self-test kits while protecting their privacy. Two participants who used to be shy about discussing HIV described the following:

Because from the MSM community, some of us are not very comfortable of getting [HIV] test kits on site, because like...fear of the stigma, that the society will judge.

Interview D

I can directly book the test kit through the chatbot, which is very useful and informative…my identity will remain anonymous, so people don’t know me.

Interview E

Participants deemed the AI chatbot useful and expressed their willingness to recommend the AI chatbot to others. Some participants suggested that the chatbot should be promoted among MSM who frequently use social networking apps, such as Grindr, Hornet, and Blued, to find sexual partners because those MSM were at higher risk for HIV and had greater need for HIV information. A participant stated the following:

I have the impression that anyone would actually need it [HIV testing]. But if we look at it from another angle, people on hookup apps like Grindr have a high tendency to hook up using those apps compared to those who don’t use them...we need to introduce the chatbot to them because...they...[have] been highly exposed [to HIV].

Interview G

## Discussion

### Principal Findings

The feasibility and acceptability of leveraging AI chatbots to promote HIV testing and PrEP among MSM in Malaysia is high. Discrimination and stigma toward HIV and AIDS are major barriers for MSM to access high-quality HIV testing and prevention services in Malaysia, and they are also primary facilitators for MSM to seek health information via internet-based platforms. Our AI chatbot prototype provides a platform for MSM to order free HIV self-test kits in an MSM-friendly environment and to empower them with resources and instructions. MSM who prefer to interact with health care providers in person can also locate HIV testing clinics or PrEP clinics through the AI chatbot. MSM highlighted these functions of the AI chatbot as very useful.

Similar to other studies, AI chatbots were well received by users [28,29]. An AI chatbot could enhance engagement with the key population [30]. As contemporary social patterns increasingly involve the integration of AI into everyday routines, AI chatbots could contribute to delivering precise details regarding HIV testing to individuals actively seeking such information. A chatbot named Eli, developed by the United Nations Educational, Scientific, and Cultural Organization, received highly favorable user feedback and was widely acclaimed [29]. Eli offers a range of services, including details on HIV prevention, testing, and treatment and assistance in overcoming fears and concerns. Compared with Eli, our AI chatbot did not have information on treatment for AIDS and provided limited mental health support. Integrating these functions into our AI chatbot may support its usability. Nevertheless, our AI chatbot offers free HIV self-test kits and locates local clinics in Malaysia for HIV testing, PrEP consultation, and mental health care.

From our previous formative research, we know that factors facilitating the acceptance of an HIV prevention AI chatbot include providing useful information and having the capacity to solve problems [13]. In this study, participants reported that our AI chatbot was able to provide useful information and help solve problems. This was indicated by the results that all participants perceived this chatbot as a helpful tool, and most participants deemed the chatbot a reliable source of information with a high satisfaction score. However, one area that required significant improvement in the chatbot was its conversation flow, as only 36% (5/14) of the participants felt that their interaction with the chatbot resembled a natural conversation. This was similar to another study where the quick response of the chatbot was deemed not humanlike and perceived as a disadvantage [28]. To address this issue and advance the chatbot, improving its algorithm and continuing training it using AI and machine learning techniques based on feedback from a larger sample size is crucial. Considering that the use of AI chatbots in health care is still in its early stages, this finding holds particular significance for designing AI chatbots. To enhance usability and promote the implementation of AI chatbots in health care, the chatbots must possess the ability to initiate natural conversations with humanlike characteristics. In addition, they should be equipped to effectively address users’ questions and concerns while ensuring the security and safety of users’ information.

The chatbot’s plain interface and simple design were popular among MSM. Digital health interventions are useful, but knowing how to navigate a digital system sometimes could be daunting for users. Through this study, we are clear that accurate and simple responses without errors and redundant information were key to the acceptability of AI chatbots among MSM. Our participants reported that the AI chatbot helped them avoid societal stigma and protected their privacy, which increased their acceptability of using the chatbot to test for HIV. This finding is consistent with our previous formative research finding that addressing sociocultural barriers can facilitate the acceptance of an AI chatbot [13]. The chatbot does not require users to provide registration information. Therefore, it can maintain participants’ anonymity. However, it is still necessary for the chatbot to clarify to users that the backend researchers and engineers who have access to users’ conversations and information will not expose users’ information to others. This suggestion is consistent with our study findings and some previous studies showing that mHealth interventions could improve HIV testing rates if users’ anonymity were guaranteed [14,17].

Some technical-related issues negatively affected the participants’ experience of navigating the chatbot. The inconvenient address input process and repeated steps owing to incomplete information contributed to the inconsistency and complexity of the chatbot, prompting participants to seek technical assistance. Many of these resulted from cultural differences, as the address options were designed based on overseas settings. This signifies the importance of tailoring the chatbot to the local context to improve usability. In addition, using localized language could also enhance the participants’ satisfaction with the chatbot. Despite the challenges inherent in adopting novel technology, the advantages of using chatbots to connect with high-risk populations could significantly impact the efforts to address public health emergencies.

In line with other studies conducted in Malaysia, MSM are keen to peruse the information on PrEP and mental health, particularly the information on where the PrEP and mental health clinics are located [14]. Most participants in our study felt that they would like to see more information through the chatbot introducing AIDS, its treatment, mental health issues, and sexually transmitted infections to better understand and manage AIDS, including how to prevent high-risk behaviors and where to seek timely help [12]. In Malaysia, professional and MSM-friendly care for mental health needs to be developed as most MSM reported that culturally sensitive information and resources regarding mental health issues were difficult to obtain. Interestingly, researchers have identified several obstacles to the adoption of AI chatbots for mental health care among users [31]. These include concerns related to privacy risks, restricted conversational engagement, negative user perceptions of personality traits (such as rudeness, lack of empathy, patronization, and being judgmental), and a lack of trust in the app’s creators. Nevertheless, Eli chatbot overcame all these challenges by having a language that merges expertise and respect for the user, ensuring speech that is gender neutral and devoid of stigmatizing elements [29]. Our AI chatbot also had a similar language as Eli, which warrants future support on mental health issues.

To increase the use of the AI chatbot, it needs to be embedded in social media platforms that MSM frequently use. The geosocial networking apps where MSM find sexual partners, such as Grindr, Hornet, and Blued, and websites owned by nongovernmental organizations and MSM-friendly clinics are important venues to advertise the AI chatbot. MSM preferred these platforms because they are trusted and frequently used by MSM. Dissemination of the AI chatbot should be promoted among young MSM who use geosocial networking apps to find sexual partners because they are at a higher risk for HIV. Through this study, we found that to embed an AI chatbot into an internet-based platform for health promotion, researchers and engineers must consider the platform’s characteristics, including its target population, level of privacy, and user-friendliness. Findings from this study will be used to improve the AI chatbot before testing on a larger scale through a national observational study in Malaysia. AI chatbots are a promising tool for promoting HIV testing and prevention. The AI chatbot must be made visible to MSM to increase its usability among MSM. Adopting the right dissemination strategies is key to increasing the visibility of AI chatbots and bringing significant impact to the MSM community. In addition, it is important for researchers to consider the sustainability of AI chatbots for MSM care in a context where sex-same sexual behaviors are criminalized. The policies and laws in Malaysia pose significant challenges on the sustainability of leveraging AI and securing funding for MSM care research. In such a political environment, it is crucial for researchers to collaborate with local nongovernmental organization and MSM-friendly clinics that operate within the existing Malaysian legal framework. Future research should focus on developing innovative and culturally tailored AI interventions to combat HIV among MSM, promote public health in Malaysia, and advocate for changes in discriminatory policies and laws to enhance the testing, implementation, and sustainability of these AI interventions.

### Limitations

Testing the AI chatbot among its end users (ie, MSM) was an important step in determining its feasibility and acceptability in Malaysia and collecting feedback to improve the chatbot further. Although this study contributed important scientific knowledge, it had several limitations. One of the limitations is that we only included MSM who can read English, as the AI chatbot is currently only available in this language. Thus, the reach of the AI chatbot may be limited only to those fluent in English, which is not the case for most MSM in Malaysia. Therefore, our findings may not be generalizable to MSM who cannot read English. Considering that Malaysia is a multilingual country with Bahasa Malaysia as the official language, the chatbot must be improved to communicate in Bahasa Malaysia or Mandarin to reach a wider audience and promote greater access to HIV self-testing and PrEP. In addition, our participants were highly educated; this may lead to bias as they might possess a certain level of knowledge and health literacy, thus facilitating their interactions with the chatbot. Therefore, the findings may differ in the less educated or literate group. In addition, our study only included MSM aged ≥18 years; therefore, the study findings do not capture the perceptions of younger MSM who are typically more tech-savvy and susceptible to HIV. Although obtaining consent from younger MSM in Malaysia for HIV-related research is a significant challenge, future studies should consider conducting surveys and interviews with MSM aged <18 years who can provide insights into the experiences and needs of the younger MSM.

### Conclusions

The AI chatbot was found to be feasible and acceptable among MSM, highlighting features, such as being informative, being able to respond to users’ questions, and having a simple and user-friendly interface. Adapting the AI chatbot to local cultures, including support for other languages, and providing additional information such as mental health support, risk assessment for sexually transmitted infections, AIDS treatment, and the consequences of contracting HIV would contribute to the successful implementation and dissemination of the AI chatbot in Malaysia.

## Appendix

Multimedia Appendix 1 An email that the research assistant sent to participants introducing the human-chatbot interaction.

Multimedia Appendix 2 The list of beta testing tasks.

Multimedia Appendix 3 The guide on chatbot beta testing.

Multimedia Appendix 4 The scales used for measuring outcome variables.

Multimedia Appendix 5 Participants’ insights with illustrative quotes.

Multimedia Appendix 6 Screenshots demonstrating the chatbot’s simple conversation flow.

## Abbreviations

AI: artificial intelligence
mHealth: mobile health
MSM: men who have sex with men
PrEP: pre-exposure prophylaxis
UTAUT: Unified Theory of Acceptance and Use of Technology
