# Supplementary material for: Testing the Feasibility and Acceptability of Using an Artificial Intelligence Chatbot to Promote HIV Testing and Pre-Exposure Prophylaxis in Malaysia: Mixed Methods Study
Source: JMIR Hum Factors. 2024 Jan 26;11:e52055. doi: 10.2196/52055 (PMC10858413; doi:10.2196/52055)
Supplement: Multimedia Appendix 1 [file humanfactors_v11i1e52055_app1.pdf]

Dear [Participant's name]:

I look forward to meeting you.

In tomorrow's meeting, we will be using a research method called think-aloud to test the chatbot. Specially, we will give you the opportunity to select three tasks to complete, such as ordering an HIV self-testing kit, and observe your interaction with the chatbot. Throughout the entire interaction process, we kindly ask you to vocalize your thoughts and imagine that you are alone.

The following statement described what is the think-aloud method:

The think-aloud method is a technique in which participants verbalize their thoughts while performing tasks, as if the researchers were not present. It is important to refrain from seeking assistance from the researchers during the testing. In case you pause or remain silent, the researchers will kindly remind you to continue thinking aloud. Please keep in mind that the chatbot is the one being tested, not you.

If you are further interested in this method, you can watch the following video:

https://www.youtube.com/watch?v=BwpPIiBK0cA

Thanks,

[Researcher's name]
