# Supplementary material for: Testing the Feasibility and Acceptability of Using an Artificial Intelligence Chatbot to Promote HIV Testing and Pre-Exposure Prophylaxis in Malaysia: Mixed Methods Study
Source: JMIR Hum Factors. 2024 Jan 26;11:e52055. doi: 10.2196/52055 (PMC10858413; doi:10.2196/52055)
Supplement: Multimedia Appendix 2 [file humanfactors_v11i1e52055_app2.pdf]

The List of Beta Testing Tasks

HIV testing

- Task 1:
  - Order a free HIV self-testing kit through the chatbot.

- Task 2:
  - Imagine that you want to test for HIV. Please interact with the chatbot to find a clinic near you that can help you test for HIV.

- Task 3:
  - Please interact with the chatbot to find out whether you need to test for HIV.

- Task 4:
  - Find a clinic that can provide HIV testing service in Kuala Lumpur.

PrEP

- Task 5:
  - Imagine that you want to take PrEP, a medication that prevents HIV transmission. Please interact with the chatbot to find a clinic near you that provides PrEP.

- Task 6:
  - Imagine that you are not sure if you need to take PrEP. Please interact with the chatbot to find out the answer.

- Task 7:
  - Find out the monthly cost of taking PrEP.

Depression

- Task 8:
  - Find out the most common symptoms of depression through the chatbot.

- Task 9:
  - Imagine that depression is your concern. Please interact with the chatbot to find out a clinic near you that can provide consulting service.

- Task 10:
  - Imagine that you want to know whether you have depression. Please interact with the chatbot to find the answer.
