# Supplementary material for: Testing the Feasibility and Acceptability of Using an Artificial Intelligence Chatbot to Promote HIV Testing and Pre-Exposure Prophylaxis in Malaysia: Mixed Methods Study
Source: JMIR Hum Factors. 2024 Jan 26;11:e52055. doi: 10.2196/52055 (PMC10858413; doi:10.2196/52055)
Supplement: Multimedia Appendix 3 [file humanfactors_v11i1e52055_app3.pdf]

The Guide on Chatbot Beta Testing

1. What is beta testing?

Beta testing is a form of user acceptance testing where the research or product team evaluates the performance of a nearly finished product among target users in the real-world conditions.[1]

If you are interested in learning more about beta testing, please read this article "*Everything You Need to Know About Beta Testing*".

2. How will we conduct a beta test?

Every beta test has 3 steps:

- 1. Chatbot-human interaction (45-minutes)
  - In this phase, we will use the think-aloud method to observe a participant's interaction with the chatbot. Specifically, every participant will be asked to share their screen on Zoom. Later, the research team will send the participant a URL to the chatbot and a list of chatbot tasks. Some examples of the tasks are listed as follows:
    - Order an HIV self-testing kit.
    - Find a clinic that can provide HIV testing service in Kuala Lumpur.
    - Find out the common symptoms of depression.
- 2. Semi-structured interview (35-minutes)
  - In this phase, the Yale team will hand over the microphone to a MIST (Malaysian Implementation Science Training) scholar. The MIST scholar will lead the interview for 35 minutes. The purpose of the interview is to ask feedback from the participant regarding his interaction with the chatbot in the phase 1. Please see the Semi-Structured Interview Guide on the following page.
- 3. Online survey (10 minutes)

The Semi-Structured Interview Guide

1. Interviewer:

- MIST scholars

2. Interviewees

- Men who have sex with men (MSM) recruited in Malaysia.

3. The Overarching Research Questions

- How was your experience of interacting with the chatbot?
- What information or features do you think need to be added to the chatbot?

4. Questions that you may ask during the interview

- How was your experience with the AI chatbot?
- What feature of the AI chatbot do you like the most?
- Is there any feature of the AI chatbot that you don't like?
- What information needs to be added to the AI chatbot to increase its popularity among MSM?
- What feature needs to be added to the AI chatbot to increase its popularity among MSM?
- What features of the AI chatbot decreased your acceptance of the chatbot?
- What features of the AI chatbot could reduce its popularity among MSM?
- YOU ARE ENCOURAGED TO ASK ANY OTHER RELEVANT QUESTIONS THAT PERTAIN TO THE TWO OVERARCHING RESEARCH QUESTIONS.

5. Section One: Establish good rapport with the participant (1 minute**)**

Framing: Thanks for your participation.

- Self-introduction.
- The purpose of this interview is to <u>understand your experience of interacting with the artificial intelligence chatbot.</u>
- This interview will take about 35 minutes.
- If at any time during the interview, you wish to stop using the recorder or end the interview, please don't hesitate to inform me.
- Your responses will remain confidential.

6. Section Two: AI chatbot (30 minutes**)**

Framing: Now I am going to ask you some questions to understand your experience of interacting with the chatbot.

1. **How was your experience of interacting with the chatbot?**
    - Probe: Which part of the responses from the chatbot did you like the most? Why?
    - Probe: Which part of the responses from the chatbot did you like the least? Why?
    - Probe: What features of the chatbot did you like? Why? (e.g., interface, linkage to resources from the website, design, font, color, response time)
    - Probe: What features of the chatbot do you think need to be improved? Why?
2. **Would the chatbot be useful in helping you get HIV tested?**
    - Probe: Ordering HIVST kits?
    - Probe: Giving instructions on the testing process?
    - Probe: Finding closest clinics to get tested?
3. **What other information that you would want or expect from the chatbot? (e.g., HIV treatment)**
4. **Have you used another chatbot before? If yes, can you tell us more about other chatbots?**
    - Probe: How did your previous experiences influence your experience today? (e.g., expectations)

7. Section Four: Dissemination of the chatbot (4 minutes**)**

Framing: I would like to know more about your perceptions on how this chatbot should be promoted or disseminated.

1. **Where would you expect to find a similar HIV testing chatbot?**
    - Probes: How about any website pages or geosocial networking apps?
    - Probes: Where would you prefer to find this chatbot?
2. **Do you have any suggestions to us regarding increasing the popularity of this chatbot among Malaysian MSM?**
3. **Would you recommend the chatbot to your friends? Why?**
    - Probe: Any specific considerations when recommending the chatbot to your friends? (e.g., age, past experience of HIV testing, use of geosocial networking apps)

Reference

1. Nick B. Everything You Need to Know About Beta Testing. 2019; https://xd.adobe.com/ideas/process/user-testing/everything-you-need-to-know-about-beta-testing/. Accessed August 12, 2022.
