# Supplementary material for: Testing the Feasibility and Acceptability of Using an Artificial Intelligence Chatbot to Promote HIV Testing and Pre-Exposure Prophylaxis in Malaysia: Mixed Methods Study
Source: JMIR Hum Factors. 2024 Jan 26;11:e52055. doi: 10.2196/52055 (PMC10858413; doi:10.2196/52055)
Supplement: Multimedia Appendix 4 [file humanfactors_v11i1e52055_app4.pdf]

The 10-point Rating Scale to Measure the Feasibility of the Chatbot

1. Overall quality of the chatbot

How would you rate the overall quality of the chatbot?

Not good at all Extremely good

0 ——————————— 10

2. Satisfaction scale

How satisfied were you with the experience of interacting with the chatbot?

Not satisfactory at all Extremely satisfactory

0 ——————————— 10

3. Intent to continue using the chatbot

If the chatbot were publicly available, how likely would you be to use it for your HIV-related needs?

Not at all likely Extremely Likely

0 ——————————— 10

4. Willingness to refer the chatbot to others

How likely would you be to recommend this chatbot to a friend or colleague?

Not at all likely Extremely Likely

0 ——————————— 10

System Usability Scale

For each of the following statement, select the answer that best describes your reactions to the chatbot. 1 ('Strongly Disagree') to 5 ('Strongly Agree')

I would like to use this chatbot frequently.

I found the chatbot unnecessarily complex.

I though the chatbot was easy to use.

I think that I would need the support of a technical person to be able to use this chatbot.

I found the various functions in this chatbot were well integrated.

I thought there was too much inconsistency in this chatbot.

I would imagine that most people would learn to use this chatbot very quickly.

I found the chatbot very cumbersome to use.

I felt very confident using the chatbot.

I needed to learn a lot of things before I could get going with this chatbot.

Adjusted Chatbot Usability Scale

The scale consists of 10 questions, scored through a five-point Likert scale from 1 ('Strongly Disagree') to 5 ('Strongly Agree')

1. It was easy to find the chatbot on the webpage.
2. My interaction with the chatbot felt like natural conversation.
3. The chatbot was able to accurately understand my input (e.g., my questions or statements).
4. The chatbot gave me too much information
5. The chatbot responded to questions too quickly, making the conversation appear unnatural.
6. The chatbot's responses were easy to understand.
7. The chatbot was helpful.
8. The chatbot gave me too little information.
9. The chatbot's responses were accurate.
10. The chatbot was slow to respond to my questions.
