# Supplementary material for: Testing the Feasibility and Acceptability of Using an Artificial Intelligence Chatbot to Promote HIV Testing and Pre-Exposure Prophylaxis in Malaysia: Mixed Methods Study
Source: JMIR Hum Factors. 2024 Jan 26;11:e52055. doi: 10.2196/52055 (PMC10858413; doi:10.2196/52055)
Supplement: Multimedia Appendix 5 [file humanfactors_v11i1e52055_app5.docx]

**Participants’ Insights with Illustrative Quotes**

| **Themes** | **Quotations** |
| --- | --- |
| **Domain 1: Performance expectancy** | |
| Reliable source of information | - Information provided is accurate, trustworthy, and comprehensive^a^ - “I can actually trust, trust this chatbot more than I can trust the Internet.” - “…if I just use this app (chatbot), I will straightaway believe…they (the researchers) have done their research, so I will have my trust on this website.” - “…everything is there, everything is informative...” - “…not too little or too much. Just nice.” |
| HIV self-testing | - The information on HIV self-testing is very helpful^a^ - “I like the fact that it gave me quick and informative pieces regarding HIVST (HIV self-testing) when I checked the HIV testing option and that it gave me options for delivery to my home or to go to the nearest clinic.” - “…it never crossed my mind that you can do HIV self-test just using it (test stick) over the gums and without blood...” |
| Facility-based HIV testing | - Information on clinics providing HIV testing is useful^a^ - “That was beyond amazing … they give [me] the addresses, contact numbers. So, I would say, if a person really needs to do the [HIV] testing, essential information like that would be very useful, so I think it’s more than helpful. The options [of HIV testing clinics] are not just [limited to] one or two [clinics], you know, so that’s good.” - Added value of human interaction^a^ - “…the reason why [they] went to a health care facility was [that they] can have someone tell them that you know, ‘Being tested positive for HIV [was] not the end of the world. To reduce the [HIV] symptoms, to reduce the [HIV] effect, to reduce [their HIV] viral load … [they] can still live a normal life and so on,’ which might be something that the self-test kits [were] lacking.” |
| PrEP-related information | - Information regarding PrEP could promote PrEP uptake^a^ - “…Because not everybody knows about PrEP including me…now I understand PrEP was to prevent from this thing (HIV), to prevent from bad things (HIV Transmission), so yeah I like it.” - “A lot of people [MSM] are always asking themselves ‘Should I get PrEP? Am I at risk? Do I need to take PrEP as a precautionary measure?’… So these are [questions] that MSM usually a bit too scared to ask the doctors.” |
| Mental health information | - Lack of information on mental health issues^b^ - “The website did not have links to any information regarding mental health issues and that is a glaring issue for it to be left out like that.” - “I just find that for the mental health, it's kind of short.” |
| Preferred features | - Preferred to have information on HIV statistics^a^ - “…maybe can include some…studies…how many percent of MSM who doesn't know that they have HIV and who have HIV…” - An instructional video should be provided alongside HIV self-testing kits^a^ - “If the self-test kit has got like an instructional video or something like that to kind of guide the users along the way of getting [themselves] tested, I think that'd be great because not everyone knows how to use a kit successfully.” - Preferred to receive information on antiretroviral therapy^a^ - “I think [providing more information about] HIV treatment would [be] very helpful because those who might be exposed to HIV would definitely want to know what the treatment is all about.” - Preferred to receive more information on HIV risk assessment^a^ - “…it didn’t tell me my risk level...” - Preferred to have information on complications of HIV^a^ - “… information on the more commonly recurring sexual transmitted diseases (STDs) will be something of beneficial to be added on…a lot of people don't exactly have that much idea on [STDs] so it kind of puts them at a higher risk.” - “…HIV and STDs…[are] not the same, but…I thought [they were] the same…I thought HIV and STDs were not curable…so I think it will be great if you can add STDs [to the chatbot].” |
| **Domain 2: Effort expectancy** | |
| Design features | - Satisfied with the chatbot design and interface^a^ - “…the information was good and straight to the point with simple videos to make it easy to understand.” - “very direct questions and direct answers.” - “Everything was smooth and nice.” - “white and blue colors [are] neutral, and it [the chatbot] takes into account [of] color blindness as well, so that's great.” - “When [the chatbot] come[s] up with three options like that, I can explore myself… I would say that [the chatbot] is more precise, it gets to the point directly.” - “I think it's good because yeah, the features are simpler and specific. I like it.” - “I think it's very user friendly.” - Able to cater users with different levels of communication skills^a^ - “As we all know, some of us didn’t have the skills to communicate, so I think…[the] chatbot…will definitely help. I think it was great.” - The design features can be further improved^b^ - “I think all the design features can be worked on further as it feels like it (the chatbot) is not polished or finished as it [should be].” - “…everything is tightly together with very little gap…there should be proper spacing…” - “The text is not properly centered in some of the boxes, and I feel like it could [be] a better design to make it look more professional.” |
| Tailoring to local context | - Use of local language added personal touch and made the chatbot more humanized^a^ - “So that was one instance of Manglish use, which is quite interesting. It's refreshing because ideally, we would want to be as human as possible. And not so robotic in its responses… a nice touch to make someone feel slightly comfortable.” - “The impression that this chatbot… probably comes from America. It’s in English, so the moment it puts up a Malaysian style saying “Boleh”... I’m very amused with this [style].” - In Malaysia, people expressed a greater level of comfort in providing their complete address^b^ - “…when the chatbot was asking for our address, I think the chatbot can say like can I get your full address or something. No need to be like one by one…” - “In Malaysia, we don't use the term “line address” or “street address”. We usually enter the full address, with the postcode and then city and state. The one on the chatbot seems to be how addresses are filled in the United States. That part needs to be tweaked slightly based on Malaysian cities.” - Choices of clinical locations needed to be tailored to local conditions^b^ - “I think depends on the size of the state…we don't have to call out (provide choices for) all the districts because Perlis is already small enough and I think…people can just go easily from one place to another in Perlis. But if…it is a big state…we need to divide it using district.” |
| Response | - Quick and friendly responses^a^ - “… [I] got a quick response [from a chatbot].” - “Sometimes as much as you would like to have a human responding to your questions, a chatbot may be your next best thing if let's say you don't want to feel judged and having responses which doesn't seem too robotic.” - “It is not too slow or not too fast. It’s in between.” - Some participants felt the chatbot responses were a bit too fast^b^ - “…the responses were a bit fast. ” - “I couldn’t fully read and it looks a bit rushing because it tells everything.” |
| Preferred features | - To expand the availability of the chatbot, it is recommended to provide it in other languages^a^ - “…perhaps to have another option of language...I think that would be able to cover more people within the local population.” - Preferred to have an option for simplified HIV self-test kit ordering process^a^ - “…too long a list of selections to go through before I could order the HIV self-tests…especially if it was a repeat order…” |
| **Domain 3: Facilitating Conditions** | |
| Linkage to more resources | - Linking this chatbot to other resources enhances its usability^a^ - “When you interact with it (the chatbot), it throws out links to you, it’s easier to navigate to the particular links from there.” |
| Pandemic influence | - Minimizing human interaction during pandemic^a^ - “…because now it’s COVID, everyone is doing it in IT (information technology) format. Having an AI chatbot is definitely much more convenient than meeting people…” |
| Promotion platform | - Chatbot should be disseminated through various platforms^a^ - “Perhaps in the website of the MAC Malaysian AIDS Council or the websites of related NGOs. Indirectly, the staff themselves gave the introduction of this app to the MSM group.” - “…if we play our Facebook, Twitter, Instagram, or YouTube, there are always mini advertisements, so who knows, [whether we] can add this [AI chatbot]? … I need to know about this [chatbot], and I hope this [chatbot] is not a scam.” |
| **Domain 4: Social influence** | |
| Stigmatization and privacy concerns | - Stigmatization is a significant concern among MSM, and the chatbot is helpful in mitigating HIV stigmatization.^a^ - “…this topic [HIV] is quite sensitive to most people, it will create like a negative energy around you…personally I don't go and ask people what is HIV, I will search myself maybe on the Internet...” - “Because from the MSM community, some of us are not very comfortable of getting [HIV] test kits on site, because like... fear of the stigma, that the society will judge.” - Self-testing information and self-testing kits provided are helpful in protecting MSMs' confidentiality compared to facility-based HIV testing^a^ - “I would be in a slight state of worry about actually going to health care facility in person, so having a sub test kit being ordered and sent to my address would be helpful.” - “I can directly book the test kit through the chatbot, which is very useful and informative …my identity will remain anonymous, so people don't know me. ” |
| Dissemination of chatbot | - Participants perceived the chatbot as useful and expressed their intention to recommend it to others^a^ - “I will recommend to anyone.” - “I just have the impression that anyone would actually need it.” - Populations at high risk for HIV were found to have a greater need for the chatbot^a^ - “I have the impression that anyone would actually need it [HIV testing]. But if we look at it from another angle, people on hook up apps like Grindr, they’ve a high tendency to hook up using those apps in comparison to those who don’t use it…we need to introduce the chatbot to them because…they…[have] been highly exposed [to HIV].” |

^a^Positive experiences or facilitating conditions towards the AI chatbot

^b^Negative experiences or barriers to the AI chatbot
