# Supplementary material for: Testing the Feasibility and Acceptability of Using an Artificial Intelligence Chatbot to Promote HIV Testing and Pre-Exposure Prophylaxis in Malaysia: Mixed Methods Study
Source: JMIR Hum Factors. 2024 Jan 26;11:e52055. doi: 10.2196/52055 (PMC10858413; doi:10.2196/52055)
Supplement: Multimedia Appendix 6 [file humanfactors_v11i1e52055_app6.pdf]

1.

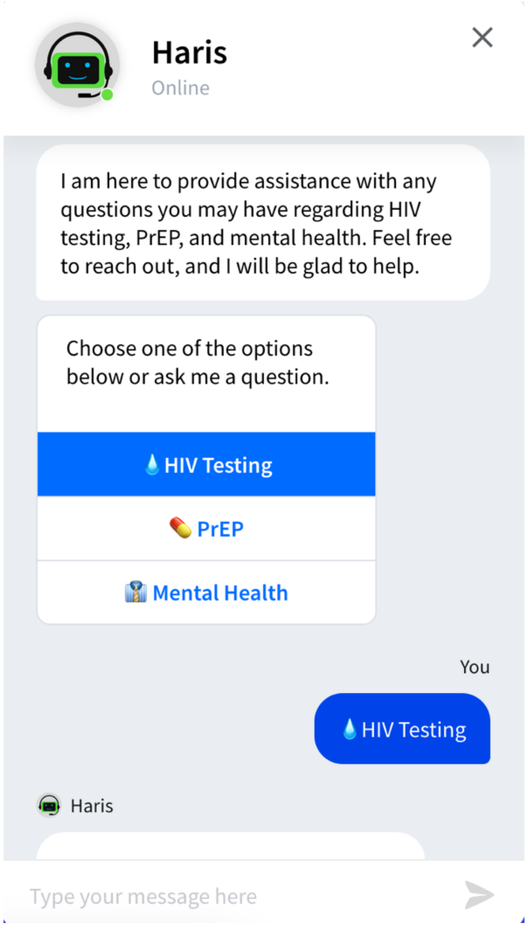
Haris
Online
I am here to provide assistance with any questions you may have regarding HIV testing, PrEP, and mental health. Feel free to reach out, and I will be glad to help.
Choose one of the options below or ask me a question.
HIV Testing
PrEP
Mental Health
You
HIV Testing
Haris
Type your message here

2.

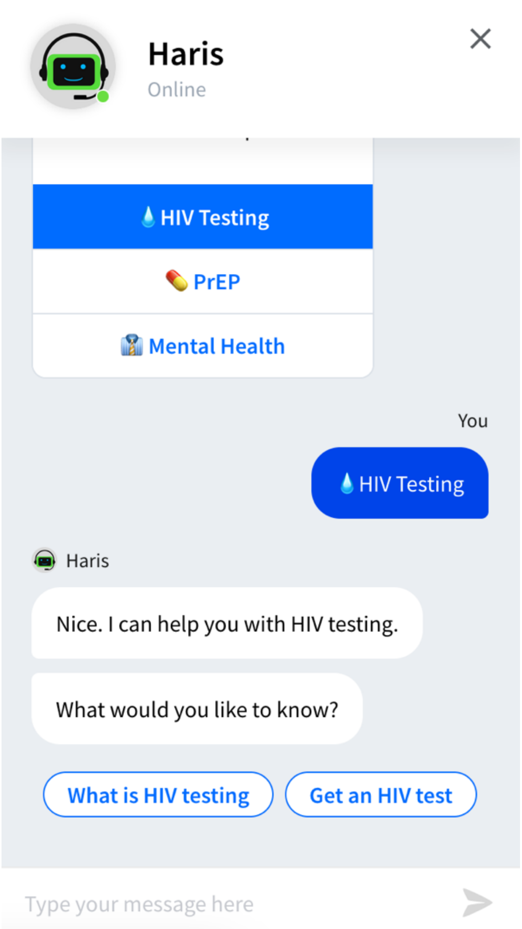
Haris
Online
HIV Testing
PrEP
Mental Health
You
HIV Testing
Haris
Nice. I can help you with HIV testing.
What would you like to know?
What is HIV testing
Get an HIV test
Type your message here

3.

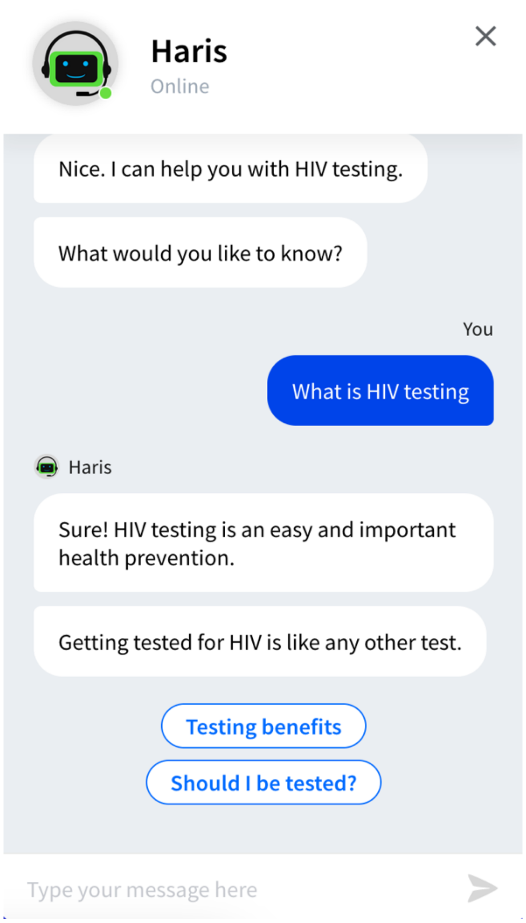
Haris
Online
Nice. I can help you with HIV testing.
What would you like to know?
You
What is HIV testing
Haris
Sure! HIV testing is an easy and important health prevention.
Getting tested for HIV is like any other test.
Testing benefits
Should I be tested?
Type your message here

4.

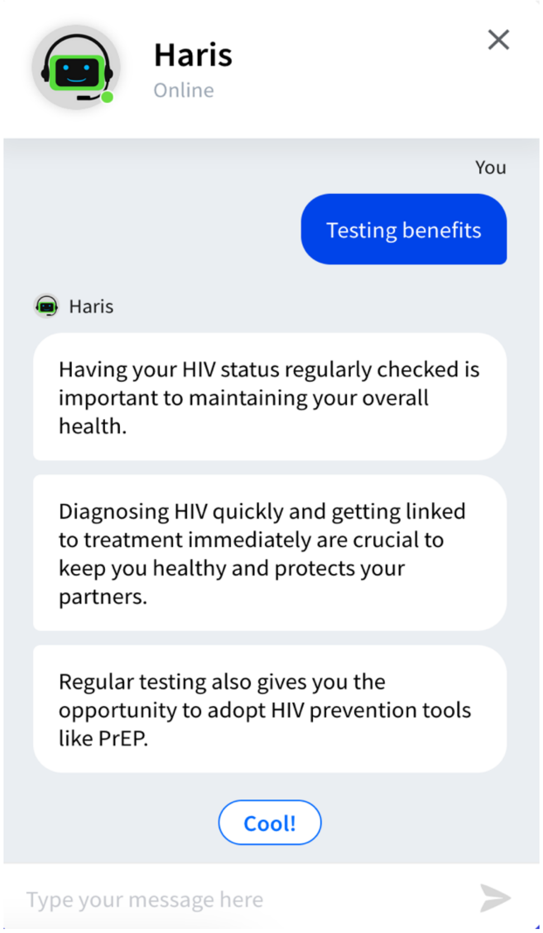
Haris
Online
You
Testing benefits
Haris
Having your HIV status regularly checked is important to maintaining your overall health.
Diagnosing HIV quickly and getting linked to treatment immediately are crucial to keep you healthy and protects your partners.
Regular testing also gives you the opportunity to adopt HIV prevention tools like PrEP.
Cool!
Type your message here

5.

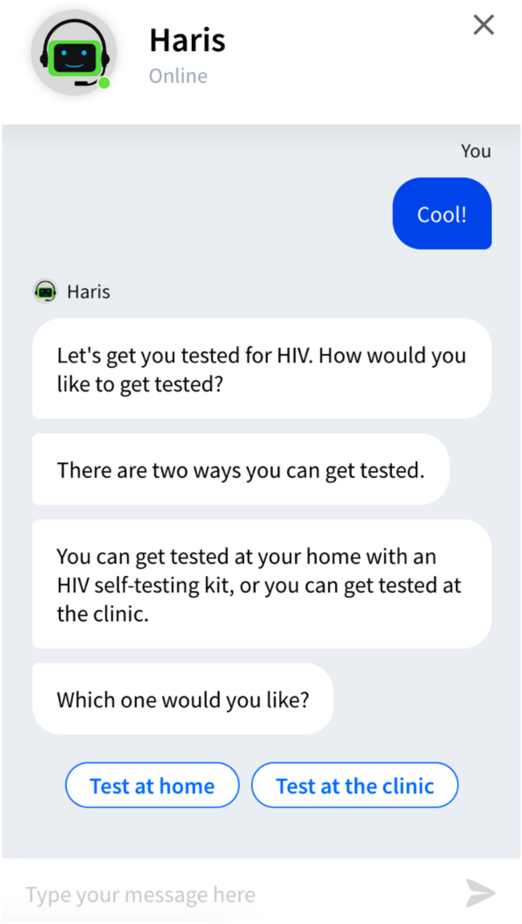
Haris
Online
You
Cool!
Haris
Let's get you tested for HIV. How would you like to get tested?
There are two ways you can get tested.
You can get tested at your home with an HIV self-testing kit, or you can get tested at the clinic.
Which one would you like?
Test at home
Test at the clinic
Type your message here

6.

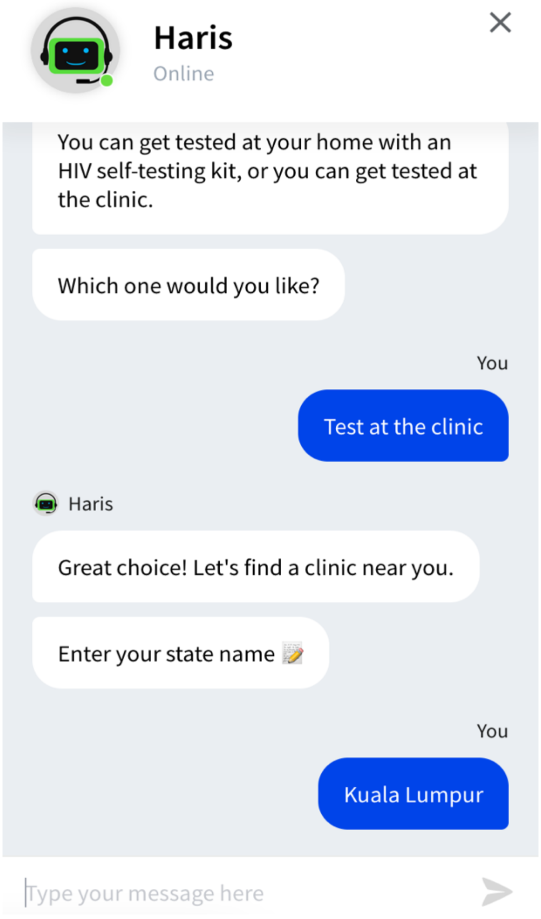
Haris
Online
You can get tested at your home with an HIV self-testing kit, or you can get tested at the clinic.
Which one would you like?
You
Test at the clinic
Haris
Great choice! Let's find a clinic near you.
Enter your state name
You
Kuala Lumpur
Type your message here

7.

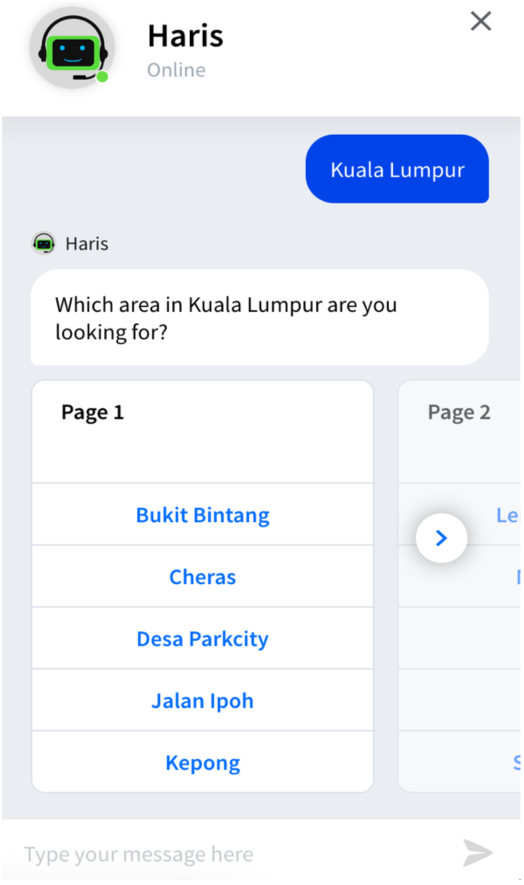
Haris
Online
Kuala Lumpur
Haris
Which area in Kuala Lumpur are you looking for?
Page 1
Bukit Bintang
Cheras
Desa Parkcity
Jalan Ipoh
Kepong
Page 2
Type your message here

8.

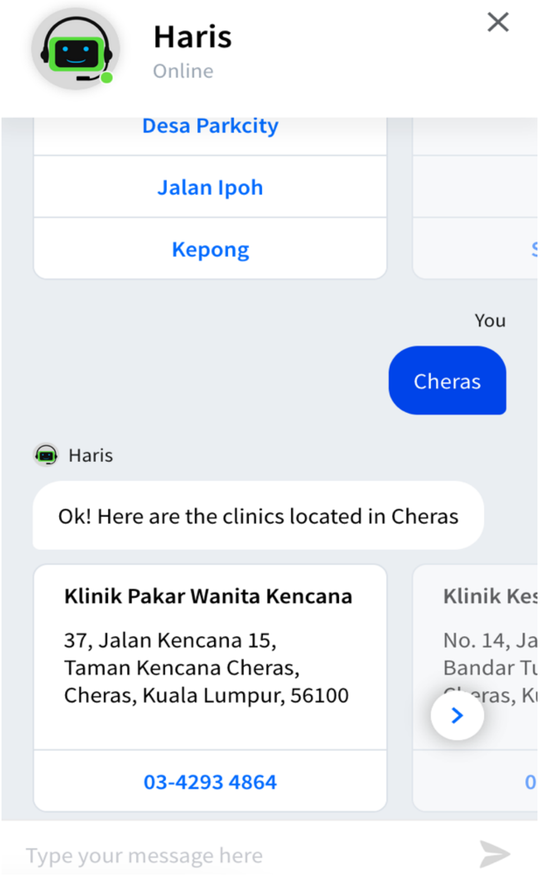
Haris
Online
Desa Parkcity
Jalan Ipoh
Kepong
You
Cheras
Haris
Ok! Here are the clinics located in Cheras
Klinik Pakar Wanita Kencana
37, Jalan Kencana 15,
Taman Kencana Cheras,
Cheras, Kuala Lumpur, 56100
03-4293 4864
Type your message here

9.

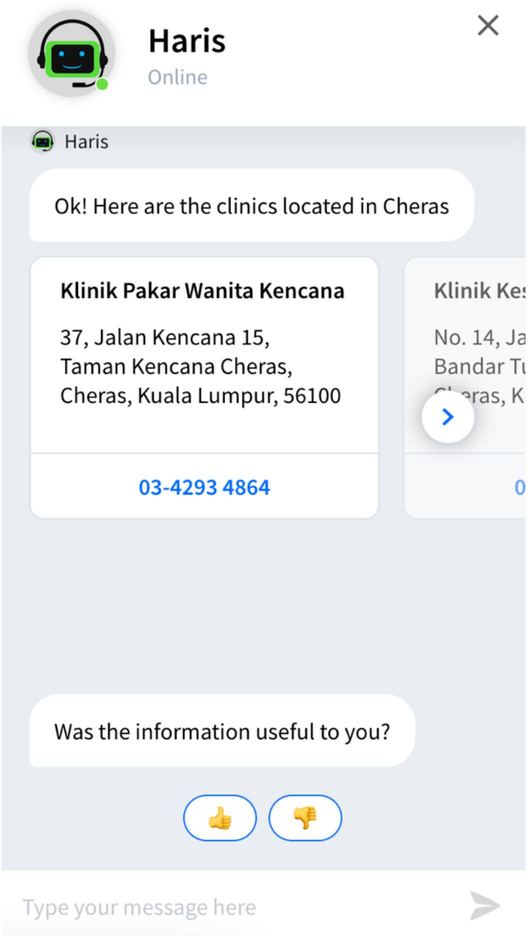
Haris
Online
Haris
Ok! Here are the clinics located in Cheras
Klinik Pakar Wanita Kencana
37, Jalan Kencana 15,
Taman Kencana Cheras,
Cheras, Kuala Lumpur, 56100
03-4293 4864
Was the information useful to you?
Type your message here

10.

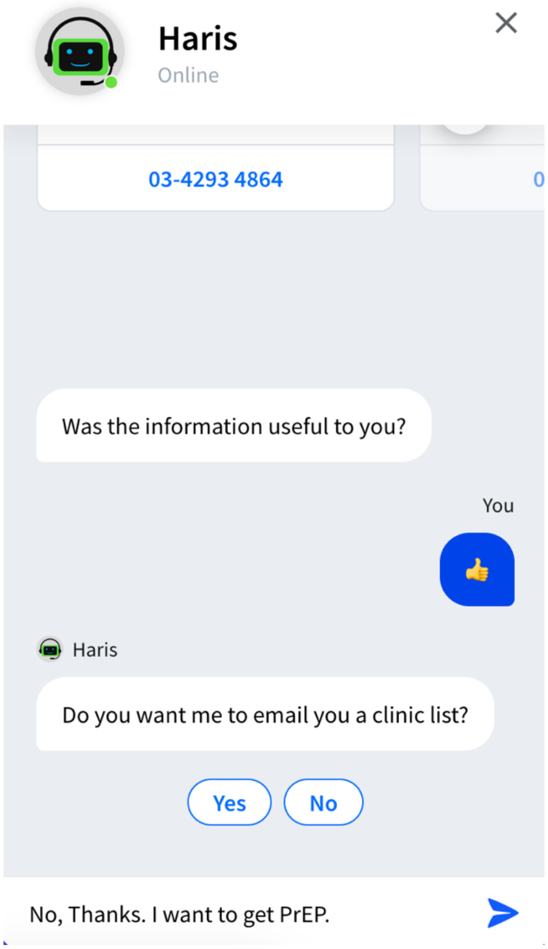
Haris
Online
03-4293 4864
Was the information useful to you?
You
Haris
Do you want me to email you a clinic list?
Yes
No
No, Thanks. I want to get PrEP.
